# Recurrent Infantile Hypertrophic Pyloric Stenosis in the Emergency Department: A Case Report

**DOI:** 10.5811/cpcem.2022.8.57140

**Published:** 2022-10-27

**Authors:** Adeola A. Kosoko, Diego Craik Tobar

**Affiliations:** The University of Texas Health Sciences Center at Houston, McGovern Medical School, Houston, Texas

**Keywords:** pyloric stenosis, vomiting, surgical failure, case report

## Abstract

**Introduction:**

Infantile hypertrophic pyloric stenosis (IHPS) is a common cause of infant vomiting. Emergency department (ED) diagnosis is usually made by pyloric ultrasound and treated by pyloromyotomy.

**Case Report:**

An eight-week-old boy with a history of IHPS about six weeks status post pyloromyotomy presented to the ED with vomiting and failure to thrive, and a critically narrowed pylorus was identified by ultrasound. An upper gastrointestinal series confirmed recurrent pyloric stenosis, necessitating another pyloromyotomy.

**Conclusion:**

Prolonged vomiting after pyloromyotomy should be concerning for recurrent IHPS. Upper gastrointestinal series should augment ultrasound to diagnose recurrent IHPS and determine whether a second pyloromyotomy is warranted.

## INTRODUCTION

Infantile hypertrophic pyloric stenosis (IHPS) is a well-described pediatric surgical emergency often presenting with “projectile” emesis of undigested milk and a hungry baby, not uncommonly with dehydration or even failure to thrive. Ultrasound has generally become more readily available in the emergency setting and has become the mainstay of IHPS diagnosis. With early ultrasound evaluation, it is less common to identify classic clinical findings such as a peristaltic wave, a palpable “olive-shaped” mass, or hypochloremic, hypokalemic metabolic acidosis. Early presentation for vomiting in a young infant, physical exam, and resultant imaging facilitate early surgical intervention and best outcomes. Although an upper gastrointestinal (GI) series may also be used to diagnose IHPS (sensitivity 100% and specificity 100%),^1^ ultrasound has become the diagnostic imaging of choice in the emergency setting (sensitivity 97–100% and specificity 99–100% with an experienced sonographer).^2^

Infantile hypertrophic plyroic stenosis is managed by increasing the size of the pyloric canal such that foods may appropriately pass. Procedures are usually curative, and recent literature suggests that recurrence of pyloric stenosis is exceedingly rare. Although some children receive balloon dilation to alleviate the pathologic obstruction of IHPS, the current mainstay of care is laparoscopic or even open pyloromyotomy.^3^ With appropriate preoperative preparation (rehydration, electrolyte repletion), pyloromyotomy is considered a relatively minor but effective and curative surgical procedure with excellent survival rates and minimal adverse outcomes.^4^ Vomiting is the most common complication in the first few days after the procedure but usually resolves with ad libitum feeds.^4^ We report the case of a young infant who had appropriately previously received surgical intervention for IHPS presenting to the emergency department (ED) with classic signs and symptoms of IHPS, diagnosed with a recurrence of IHPS by findings from an abdominal ultrasound and an upper GI series, necessitating a second pyloromyotomy.

## CASE REPORT

**Figure 1:**
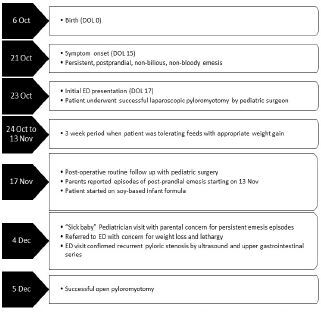
Timeline of events for an infant with recurrent hypertrophic pyloric stenosis. Day of Life (DOL), Emergency department (ED).

**Figure 2:**
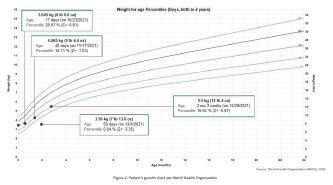
Patient’s weight-for-age growth per the World Health Organization standard statistical distribution describing boys from birth to two years old. Kilogram (kg), pound (lb), ounce (oz), month (mo).

An eight-week-old male patient presented to the ED with his parents for a three-week history of frequent episodes of post-prandial non-bloody and non-bilious emesis (Figure 1).The parents were concerned for repeated episodes of emesis for three weeks that were initially intermittent but had become consistent with feeds. Despite being advised by a pediatrician to try a soy-based formula by a telemedicine visit for presumed lactose intolerance a few days prior to this ED visit, the patient’s symptoms persisted. The parents also noticed that the patient was taking less volume when feeding. The mother described the most recent episodes of emesis as “projectile,” large volume, and comprised of what looked like his formula. In addition, he constantly seemed hungry. The parents again took him to his pediatrician where he was noted to have lost weight and seemed lethargic (Figure 2). The pediatrician recommended another ED visit.

On further interview with parents and on chart review, the patient was born at term by vaginal delivery with a birth weight of 3,460 grams and a normal physical exam. Six weeks prior, at 17 days of age, he was taken to the ED after two days of persistent, forceful, non-bloody and non-bilious emesis, which occurred after every feed. During that initial ED visit, the patient’s basic metabolic profile was normal, including a potassium of 4.6 milliequivalents per liter (mEq/L) (reference range: 3.7–5.2 mEq/L) and chloride of 103 mEq/L (reference range: 96–106 mEq/L), and an abdominal ultrasound was concerning for an abnormally large pylorus measuring 2 centimeters (cm) in length and 4 millimeters (mm) in width with no passage of food contents. The patient urgently underwent a successful laparoscopic pyloromyotomy without complication. He was discharged home with his parents with an uneventful three-week period including normal oral intake and adequate weight gain for his age.

**CPC-EM Capsule**

What do we already know about this clinical entity?

*Hypertrophic pyloric stenosis is a well-described pathology causing vomiting leading to failure to thrive in infants. Diagnosis is usually made by ultrasound*.

What makes this presentation of disease reportable?

*This presentation describes a case of a child who had already received a pyloromyotomy with another clinically classic case of pyloric stenosis*.

What is the major learning point?

*When pyloric stenosis is diagnosed very early in life, despite appropriate surgical intervention, it could possibly recur. An upper gastrointestinal series may help with diagnosis*.

How might this improve emergency medicine practice?

*The clinical signs and symptoms of pyloric stenosis are generally consistent and require appropriate evaluation even if a child has already what is typically definitive intervention*.

On evaluation, the patient was lethargic with a weak cry. The child appeared small for stated age with dry mucous membranes and a flat fontanelle. Vital signs on presentation included a temperature of 98.5° Fahrenheit, heart rate 130 beats per minute, respiratory rate 35 breaths per minute, oxygen saturation 98%, and blood pressure 81/48 millimeters of mercury (mm Hg). The patient had clear bilateral tympanic membranes and a normal posterior oropharynx. His chest was clear to auscultation, cardiac exam was grossly normal, and his abdomen was soft, without dilatation, and without palpable masses. He had a three second capillary refill (normal: <2 seconds) and a normal genital exam for his age. The child produced a weak cry when nurses started a peripheral intravenous line.

A basic metabolic panel was significant for a hypokalemic (2.6 mEq/L), hypochloremic (76 mEq/L) metabolic alkalosis. A venous blood gas showed a pH greater than 7.70 (reference range: 7.31–7.41), partial pressure of carbon dioxide 35 mm Hg (reference range: 41–51 mm Hg), and partial pressure of oxygen 53 mm Hg (reference range: 30–40 mm Hg). We were unable to calculate bicarbonate and base excess due to a pH more than 7.70. The patient underwent an abdominal ultrasound in the ED, which suggested IHPS with an enlarged pyloric channel measuring 2.1 cm and a thickened muscle measuring 0.5 cm, again with minimal passage of fluids through the pylorus (Image 1). The surgical team was consulted for the abnormal laboratory and ultrasound findings, but due to the possibility of postoperative edema or residual abnormal external pylorus measurements, the consultant recommended further imaging to conclusively determine pyloric stenosis. The child was admitted for fluid resuscitation and electrolyte replacement. An upper GI series performed the same day confirmed the diagnosis of IHPS when there was lack of contrast passing from the stomach to the duodenum. The patient received a solution of intravenous 5%, dextrose, half normal saline, and 40 mEq potassium chloride at maintenance until electrolytes and intravascular volume were optimized the next morning. The patient then underwent an uncomplicated open pyloromyotomy by a pediatric surgeon. The patient recovered well from the operative procedure and tolerated ad libitum oral feeds both in the hospital and at discharge. Three months following his open pyloromyotomy, the primary care clinic reported that the child had been tolerating feeds well and gaining weight adequately without any subsequent recurrence or complications of IHPS.

## DISCUSSION

The underlying cause of a recurrent hypertrophic pyloric stenosis after a successful pyloromyotomy remains unclear. Emesis after feeds is a common and is the most frequent complaint post pyloromyotomy. It is likely due to pyloric edema, pylorospasm, or reflux.^5^ Usually postoperative emesis will resolve spontaneously but if it persists for more than five days, incomplete pyloromyotomy should be suspected. Recurrent pyloric stenosis is a rare entity, with an incidence of less than 2% of all children who undergo successful surgery.^6^ To avoid confusion between recurrence and an incomplete surgical procedure, certain criteria need to be met. True recurrent pyloric stenosis includes complete resolution of symptoms for three or more weeks before recurrence of emesis, weight gain, and evidence of re-stenosis on sonographic or operative confirmation.^7^ The patient described in this case returned to baseline oral intake, had positive weight gain, and no episodes of emesis for three weeks before symptoms reappeared.

Ankermann et al (2002) described evaluation for recurrent pyloric stenosis. If postprandial projectile emesis occurs after successful operation and a symptom-free interval, the absence of fluid passage through the pyloric channel should be demonstrated radiologically with a swallow of water-soluble contrast before reoperation is considered.^8^ In cases of recurrent pyloric stenosis, sonographic images have to be interpreted carefully and may not be the image of choice given that the thickness and length of the pyloric channel may remain enlarged after pyloromyotomy and may not return to normal until approximately six weeks^9^ and up to five months^5^ after the procedure.^8^,^10^

Given that the etiology of recurrent pyloric stenosis after a successful pyloromyotomy is unknown, one consideration is that “recurrence” is actually a result of the progression of the original disease and the initial surgery was probably performed at an early stage after which the pylorus continued undergoing an active process of hypertrophy.

## CONCLUSION

Infantile hypertrophic pyloric stenosis is easily identified on abdominal ultrasound as a cause of refractory vomiting. Although rare, despite surgical pyloromyotomy, a minority of infants may develop a recurrence of IHPS, presenting clinically similarly to the sentinel case. Diagnosis can be made by repeated ultrasound but should be augmented by upper gastrointestinal series to avoid false positive diagnoses, which might actually be the result of postoperative edema. Management for recurrent cases is a second pyloromyotomy, which usually has good and definitive outcomes.

## Figures and Tables

**Image:**
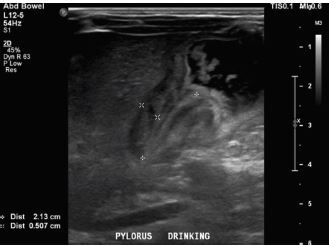
Ultrasound of pylorus with child drinking demonstrating hypertrophy (length 2.13cm and width 0.5cm) with minimum passage of fluids through the pylorus.
